# A protocol for the longitudinal investigation of cancer related fatigue in head and neck cancer with an emphasis on the role of physical activity

**DOI:** 10.1371/journal.pone.0308400

**Published:** 2024-08-14

**Authors:** Prahalad Narasimhan, Andrew R. Levy, Simon N. Rogers, Andrew G. Schache, Joanne M. Patterson, Nefyn H. Williams, Rachel C. Brooker, Adrian W. Midgley

**Affiliations:** 1 Department of Sport and Physical Activity, Edge Hill University, Ormskirk, United Kingdom; 2 Health Research Institute, Edge Hill University, Ormskirk, United Kingdom; 3 Department of Psychology, Edge Hill University, Ormskirk, United Kingdom; 4 Wirral University Teaching Hospital, Wirral, United Kingdom; 5 Faculty of Health and Social Care, Edge Hill University, Ormskirk, United Kingdom; 6 Liverpool Head and Neck Centre, Liverpool University Hospitals NHS Foundation Trust, Liverpool, United Kingdom; 7 Institute of Population Health, University of Liverpool, Liverpool, United Kingdom; 8 The Clatterbridge Cancer Centre NHS Foundation Trust, Wirral, United Kingdom; University of Study of Bari Aldo Moro, ITALY

## Abstract

**Background and aim:**

Cancer related fatigue significantly impairs the ability to undertake sustained physical activity across the domains of daily living, work and recreation. The purpose of this study is to monitor cancer related fatigue and the factors affected or caused by it for 12 months in head and neck cancer patients following their diagnosis. Their perceptions of how fatigue might affect their activity levels in addition to identifying avenues to improve engagement with physical activity will be also explored.

**Methods:**

A single centre longitudinal mixed-methods study will be conducted. Forty head and neck cancer patients will be recruited over 6 months following the confirmation of their treatment plan, after which fatigue and physical activity will be assessed at four time points over 12 months. Additionally, other factors which influence fatigue such as body composition, blood counts, systemic inflammation levels, haemoglobin concentration, thyroid function, sleep quality, cardiorespiratory fitness and upper and lower extremity strength will be measured to understand how the multifactorial problem of fatigue may evolve over time and influence physical activity levels. Semi-structured interviews will be conducted after treatment completion and at end of twelve months which will analyse the participants fatigue experiences, understand how their perceived fatigue may have impacted physical activity and report the factors which may improve engagement with physical activity during cancer. Quantitative data will be analysed and reported using standard descriptive statistics and post-hoc pairwise comparisons. The changes in outcome measures across time will be analysed using the MIXED procedure in SPSS software. Statistical significance will be accepted at p<0.05. Qualitative data will be analysed using the Interpretative Phenomenological Approach using the NVivo software.

**Discussion:**

The results from this study may help inform the planning and delivery of appropriately timed interventions for the management of cancer related fatigue.

## Introduction

### Rationale and background

Fatigue has been clearly identified and recognised as a major side effect of cancer and its treatments [1]. This fatigue is different from the fatigue experienced by the general/healthy population and is termed cancer related fatigue or cancer fatigue [2]. The National Comprehensive Cancer Network (NCCN) defines cancer related fatigue as a multidimensional syndrome characterised by a persistent, distressing and subjective sense of physical, emotional and cognitive exhaustion or tiredness associated with cancer therapy that is not proportional to the level of performed physical activity [3]. Cancer related fatigue affects physical, mental and emotional well-being resulting in an inability and/or decreased motivation to engage in activities of daily living [4]. This fatigue is one of the most distressing symptoms experienced by the patient and is a strong and independent predictor of decreased health related quality of life (HRQoL) and survival [2, 3].

On average 56–85% of all head and neck cancer (HaNC) patients report feeling fatigued to their oncologist [5, 6]. Across all types of HaNCs, 67%, 11% and 7% of patients report mild, moderate and severe fatigue respectively [6]. A recent study in the UK has identified cancer related fatigue to be the sixth highest ranked concern out of fifty six given by HaNC patients and has been shown to significantly affect the individual’s quality of life (QoL) [7, 8]. While expert oncology guidelines recommend regular screening for cancer related fatigue [9], the symptom is frequently underreported, underdiagnosed and poorly managed across the cancer continuum [3, 9]. The distinction between tiredness and fatigue is poorly made in clinical practice [3]. This, in part, maybe due to the of the increasing 5-year survival of HaNC patients, and physicians are increasingly likely to see more HaNC patients complain of chronic fatigue, fatigue related disability and suboptimal quality of life [10, 11]. Physicians may not have adequate knowledge of cancer related fatigue and its management or underestimate the severity of the fatigue and its impact on quality of life as described by the patient [12, 13]. While the recently developed Patients Concerns Inventory has been shown to be an effective tool for identifying concerns such as fatigue in HaNC patients [7], it is not yet a part of routine clinical practice across the UK National Health Service (NHS). Data from acute care trusts in the NHS also suggests that post-cancer treatment follow up care is neither universal nor consistent across the nation [14] despite 94% of oncology healthcare providers highlighting fatigue as a key problem. As a result of these factors, the timely interventions for cancer related fatigue are not being provided to HaNC patients.

Physical activity has been shown to be a feasible, effective, safe and inexpensive tool in reducing the patient’s fatigue burden in multiple cancer diagnoses [15]. However, only 9% of HaNC patients meet physical activity guidelines after their diagnosis [16]. The literature also indicates that 22% of HaNC patients are not interested in participating in any physical activity after their diagnosis. Notably, 17% of all HaNC patients report being completely unable to participate in any form of physical activity or exercise and a further 28% are unsure of their ability to participate in moderate intensity physical activity [17]. These statistics may reflect underlying fatigue and misconceptions about engaging in physical activity during cancer. While research on patient perceptions or misconceptions regarding physical activity in curable HaNC populations is lacking, the literature on palliative HaNC suggests that patients are typically advised to rest as much as possible by their consultants. Referrals to an exercise professional or physical activity counselling only took place after the transition to palliative management is finalised [18]. This late initiation of physical activity engagement is a significant issue in clinical practice as 68% of palliative HaNC patients report fatigue as a symptom or key concern relating to their quality of life [18]. Therefore, it would be safe to assume that this fatigue is not new but has been an ongoing concern throughout the cancer continuum.

Physical activity could reduce fatigue levels by improving functional capacity and cardiorespiratory fitness which helps the patient spend less effort on activities of daily living resulting in a reduced perception of fatigue [19]. Regular physical activity has also been shown to improve skeletal muscle strength, joint mobility, inflammation, anxiety, and depression in cancer survivors [20]. As these factors can directly or indirectly influence fatigue, the changes in these symptoms can affect recovery [21]. A recent meta-analysis investigating 11525 survivors of mixed cancer diagnoses also concluded that non-pharmaceutical management strategies such as physical activity are to be considered first line treatment for cancer related fatigue as they are significantly more effective than the available pharmacological treatments [22].

There is a lack of objectively measured cancer related fatigue in physical activity research conducted with HaNC patients. Majority of the physical activity/rehabilitation studies in HaNC broadly assess quality of life, where physical activity has been shown to significantly improve the patient’s short term and long-term quality of life [23, 24]. A recent report on patients with mixed cancer diagnoses suggests that many HaNC patients reported fatigue that impaired their daily activities 1-year post treatment indicating that cancer related fatigue may be a chronic health issue for these patients [25]. However, this requires further investigation as the literature on long term fatigue in HaNC is lacking. Although various clinical practice guidelines [9] recommend fatigue screening and assessment across the disease timeline along with referrals to the appropriate multidisciplinary healthcare team (MDT) professionals for its management, the current clinical practice in HaNC does not reflect this [26]. Thus, a detailed investigation into the changes in fatigue over time, perceptions of fatigue, factors affecting fatigue and the impact on physical activity within the context of HaNC is warranted.

### Study aims and objectives

There have been no detailed longitudinal investigations on cancer related fatigue and its influence of physical activity, nor have there been adequate considerations given to physical and physiological changes over time during the first 12 months of cancer. Therefore, the primary aim of this study is to describe changes in cancer related fatigue over time. The secondary aim is focused on the participants’ perception of fatigue and how it affects their physical activity during cancer. The objectives of this study are:

1. Investigate how levels of fatigue change over time from diagnosis and the following 12 months in HaNC patients.
2. Assess variables which influence cancer related fatigue such as body mass, body composition, sleep quality, physical activity levels, haemoglobin concentration, blood counts, thyroid profile, systemic inflammation, cardiorespiratory fitness, agility and upper and lower limb strength over a period of 12 months.
3. Investigate HaNC patients’ perceptions of how fatigue affects their capacity and motivation to undertake physical activity with respect to activities of daily living, work and recreation.
4. Identify patient-related factors which may improve engagement with physical activity during cancer.

## Materials and methods

### Research design and study setting

This single group longitudinal study (IRAS 308808) will take place at a regional National Health Service (NHS) Hospital, Aintree University Hospital (a part of Liverpool University Hospitals, NHS Foundation Trust). Aintree University Hospital is located in Liverpool in the North West of England and is the largest single centre HaNC unit in the United Kingdom. This is a longitudinal and observational study i.e.; no intervention will be provided as part of the research to the participants.

### Ethics

Favourable ethical opinion was given by the Leeds-West Health Research Authority Ethics Committee (Ref: 22/YH/0126, IRAS 308808).

### Participants

Forty newly diagnosed HaNC patients will be recruited from Aintree University Hospital. The samples size of n = 40 is sufficient for quantitative data analysis and is an achievable target for the study. The goal of the recruitment is pragmatic where the maximum possible participants will be invited to participate during the recruitment window via oncology multidisciplinary team referrals and from HaNC clinics at Aintree University Hospital. All participants must satisfy the following inclusion criteria: 1) ≥ 18 years old; 2) able to provide informed consent; 3) newly diagnosed with HaNC and being treated with curative intent; 4) Patients treated at, returning to, or managed at Aintree University Hospital only.

Patients who are palliatively managed, currently participating in any physical activity/rehabilitation/prehabilitation study and any patients with cognitive impairment and/or psychiatric illness will be excluded from the study. Notably, HaNC patients who are enrolled into other observational studies will not be excluded from participating. Patients enrolled in interventional studies investigating health related outcomes will be excluded from the recruitment as those interventions may affect the physical and physiological outcomes being investigated in this study.

### Recruitment

**Fig 1:**
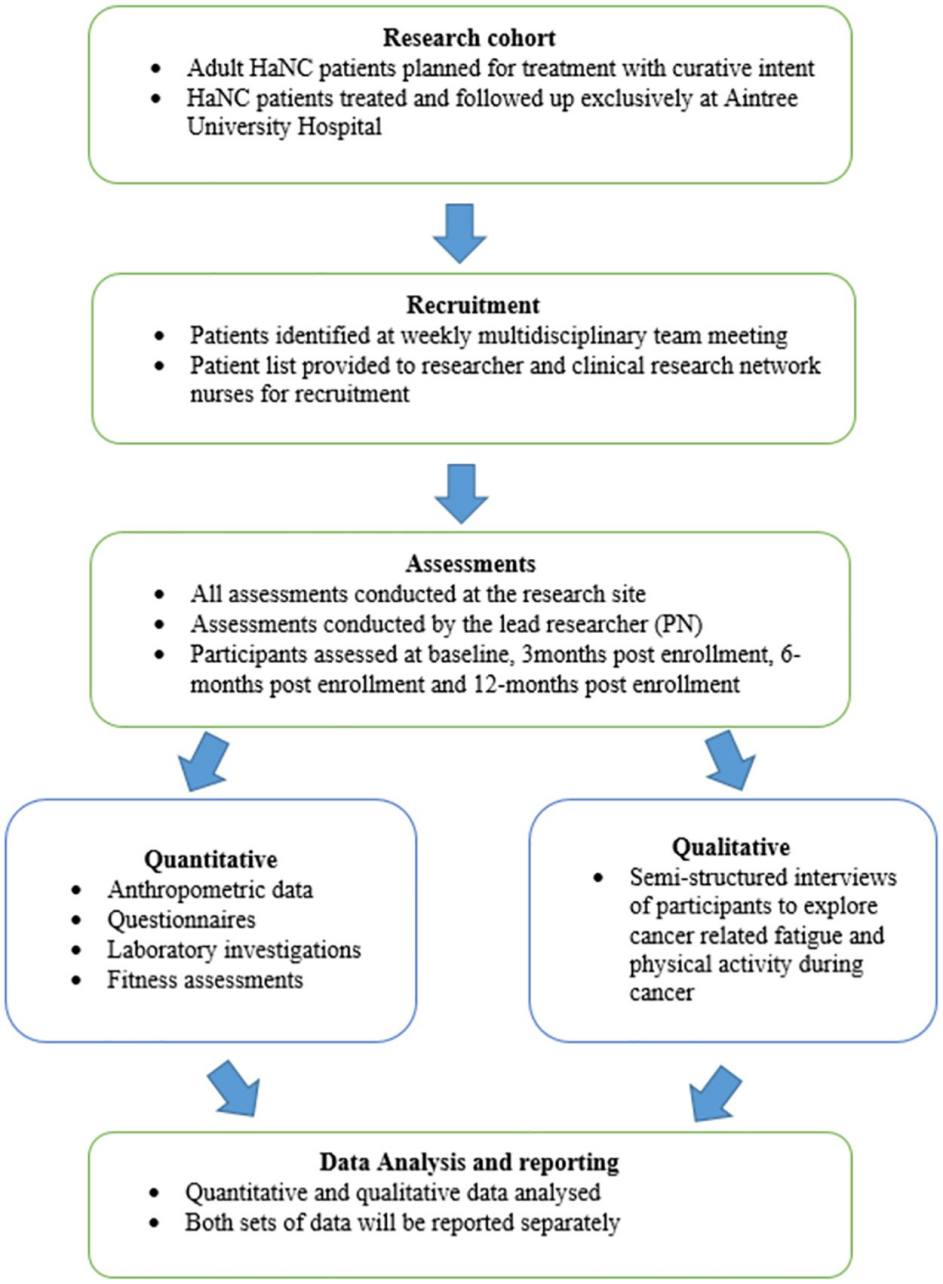
Study recruitment and pathway.

Eligible newly diagnosed HaNC patients will be invited to participate in the study with the assistance of the Clinical Research Network staff at Aintree University Hospital. Patients will be given a participant information sheet, have the study procedures explained to them, and given sufficient time to obtain answers to any questions they may have. Written and verbal informed consent for the quantitative and qualitative aspects of the study will be obtained from all participants prior to the commencement of data collection. Additionally, consent will be obtained to access their electronic medical records during the duration of the study to acquire the following information: demographics, anthropometrics, disease information, treatment information, adverse effects, and laboratory reports and imaging studies. All data will be protected from the public by pseudo-anonymising the patient post-recruitment. This will be done by using bespoke participant numbers to denote all recruited patients into the study, the details of which will be known by the lead researcher and clinical trials nurses supporting the study. Only anonymised data will be used in the analysis. The anonymisation document will be password protected and stored in accordance with all GDPR regulations with a copy available at the research site. The recruitment and study pathway is detailed in Fig 1.

### Assessment schedule and study outcomes

Newly diagnosed HaNC patients will be enrolled into the study and followed-up for 12 months after their enrolment into the study. The participants will undergo quantitative and qualitative assessments at various timepoints as detailed below in Tables 1 and 2. These timepoints coincide with the routine follow-up clinic visits for HaNC patients treated with curative intent.

**Table 1:** Schedule of assessments at the four data collection timepoints (T1-T4).

Time of assessment	Questionnaires	Fitness tests	Blood investigations	Interview	Survival and Dropouts
**T1–Diagnosis/Pre-treatment**	**✓**	**✓**	**✓**	**×**	**×**
**T2–2–3 months post enrolment**	**✓**	**✓**	**✓**	**✓**	**✓**
**T3–5–7 months post enrolment**	**✓**	**×**	**×**	**×**	**✓**
**T4–11–13 months post enrolment**	**✓**	**✓**	**✓**	**✓**	**✓**

**Table 2:** Outcomes and assessments in chronological order as recommended by the American College of Sports Medicine [27].

Assessment Tool/Modality	Domain of measure	Rationale	Timepoint
**Body Mass Index (BMI)**	Anthropometrics and body composition	Weight loss, sarcopenia and cachexia during cancer result in changing body composition. Sarcopenia and cachexia are negatively associated with prognosis in HaNC and can significantly impair physical activity, energy levels and HRQoL [28].	T1, T2, T3, T4
**Bioimpedance analysis testing (BIA testing)**			T1, T2, T3, T4
**International Physical Activity Questionnaire—Long Form (IPAQ-LF)**	Physical activity levels	Although intended for use in adults aged 18–69, the literature shows that the IPAQ-LF has moderate validity for measuring physical activity and sedentary behaviour in older adults aged 70 and above [29, 30]. The lower psychometric properties for the IPAQ-LF in older adults is due to their tendency to underreport or misremember their PA as it usually tends to be unstructured with large daily variations [29].	T1, T2, T3, T4
**Multidimensional Fatigue Symptom Inventory—Short Form (MFSI-SF)**	Cancer related fatigue	MFSI-SF has good psychometric properties with moderately strong concurrent, convergent, divergent and discriminative validity as well as excellent internal consistency and test-retest reliability [31].	T1, T2, T3, T4
**Pittsburgh Sleep Quality Index (PSQI)**	Sleep Quality	Sleep duration and quality have an impact on energy levels and recovery, with consistently poor sleep resulting in increased fatigability. The PSQI has been used and validated in cancer populations [32]	T1, T2, T3, T4
**Haemoglobin**	Anaemia	Studies have shown that compared to the general population with anaemia, cancer survivors with anaemia report greater fatigue [33].	T1, T2, T4
**Total Blood Count**	Immune function	Low red blood cell count and the impaired immune function as a result of cytotoxic cancer therapy, has been linked to the development of cancer related fatigue [34].	T1, T2, T4
**C-Reactive Protein (CRP)**	Inflammation	Fatigue is associated with inflammation in HaNC before and after intensity-modulated radiation therapy (IMRT) with an increase in CRP post IMRT positively correlated to an increase in fatigue [5, 35].	T1, T2, T4
**Thyroid Function Test (TFT)**	Thyroid function	Primary hypothyroidism as a result of cervical radiation is insidious in its onset and is one of the multifactorial causes of cancer related fatigue in HaNC survivors as hypothyroidism is shown to cause tiredness, lethargy, muscle cramps as well as peripheral oedema [36].	T1, T2, T4
**Modified Incremental Shuttle Walk Test (ISWT)**	Aerobic fitness	The ISWT is an inexpensive cardiopulmonary fitness assessment tool validated for use in cancer populations with good reliability and validity with the psychometric properties of the test valid for both younger as well as older cancer patients/survivors. The modified ISWT is a useful surrogate for cardiopulmonary exercise testing (CPET) as it is a maximal exercise test and can be used to estimate peak oxygen uptake [37].	T1, T2, T4
**Grip strength—Handheld Dynamometry**	Upper and lower body strength	Low muscular function is associated with greater morbidity and mortality with low muscular strength linked to higher all-cause mortality from cardiovascular dysfunction. Grip strength has also been shown to be inversely related with all-cause mortality [38].	T1, T2, T4
**Timed up-and-go test (TUG)**	Agility, Balance, Lower body power	The TUG test is a measure of functional mobility used to measure lower body power as well as identify agility and balance impairments in older adults. The TUG has shown to have high interrater and intra-rater reliability when used to assess elderly adults as well as older cancer patients. It has also demonstrated good validity for assessing functional mobility [39].	T1, T2, T4
**30 Second sit-to-stand test**		The 30 second sit-to-stand test is a measure of lower extremity strength and endurance assessment for older adults. The test has excellent inter-rater and intra-rater reliability and is valid across multiple clinical populations [40].	
**Semi-structured interviews**	Lived experiences of fatigue and physical activity during cancer	Semi-structured interviews will be used to gain insight into the lived experiences of fatigue and understand the perceived impact of fatigue on physical activity. In addition, HaNC patient driven factors to improve engagement with physical activity will be identified.	T2, T4

**HaNC** = head and neck cancer; **HRQoL** = health-related quality of life.

Participants will undergo pre-exercise health screening prior to commencing the fitness assessments at any given timepoint. If any absolute contraindications are observed, the participant will not be allowed to participate until the problem has been resolved in consultation with their oncology care team. Contraindications to participating in the fitness assessments are described in S1 File.

### Blood sampling and analyses

Blood samples will be collected from the participant at the hospital by a registered nurse prior to all physical tests via venepuncture. Two samples of approximately 5ml each will be collected at each of the relevant assessment timepoints (T1, T2 and T4). Taking blood samples before physical activity avoids haemoconcentration from plasma moving out of the blood circulation due to increased systolic blood pressure and from fluid loss due to sweating during physical activity, which causes changes in the measurement values [41]. Collected samples will be labelled with the participant study number and/or their NHS number then transported to the Aintree University Hospital laboratory for appropriate storage (at 2-8^0^ C) and processing. Reports will be generated by the laboratory and provided to the study PI or the researcher.

#### Full blood count (FBC)

FBC is a group of tests which are assessed via an automated FBC assay which provides haemoglobin concentration, red cell indices, white blood count (with differential counts) and platelet counts in addition to mean platelet volume and meancorpuscular volume. The sample will be collected in a standardised EDTA (Ethylenediamine tetraacetic acid) blood test-tube which prevents clotting of the sample. A standard FBC takes 4–12 hours. Normative values for the component measures differ by gender.

#### C-reactive protein (CRP)

CRP will be assessed using a standardised Tina-quant C-Reactive Protein IV test using a Cobas C 701/702 analyser (Cobas^®^, Indianapolis, USA) via a 2-point end assay using either Tris(hydroxymethyl)-aminomethane buffer with bovine serum albumin (R1) or latex coated with anti-CRP (mouse) in glycine buffer (R3) reagents. CRP agglutinates with latex particles coated with monoclonal anti-CRP antibodies and the aggregates are determined turbidimetrically. Cobas C analysers automatically calculate the analyte concentration of each sample. A CRP level of less than 0.3 mg/dL (milligrams per decilitre) is considered normal in healthy adults.

#### Triiodothyronine (T3)

T3 will be assessed via Elecsys FT3 III assay using a Cobas e411 analyser (Cobas^®^, Indianapolis, USA) where a specific anti-T3 antibody labelled with a ruthenium complex is used to determine the free triiodothyronine concentration. Testing comprises of an 18-minute assay with two incubation periods in between where the sample is mixed with anti-T3-specific antibodies, biotinylated T3 and streptavidin coated microparticles. The reaction mixture is aspirated and the microparticles are magnetically captured onto the surface of an electrode. Application of voltage to this electrode induces chemiluminescent emissions which is then measured by a photomultiplier and the final result estimated using a instrument provided calibration curve. A T3 level of 3.1–6.8 pmol/L (picomoles per litre) is considered as normal.

#### Thyroxine (T4)

T4 will be assessed via the Elecsys FT4 III assay using the Cobas e411 analyser where a specific anti-T4 antibody labelled with a ruthenium complex is used to determine the free thyroxine concentration. The 18-minute assay follows the same steps similar to T3 except anti-T4-specific antibodies are used and the final results estimated using an instrument provided calibration curve from the chemiluminest emissions. Both T3 and T4 assays take 18 minutes to complete. A T4 level of 12–22 pmol/L is considered normal.

#### Thyroid stimulating hormone (TSH)

TSH will be assessed via the Elecsys TSH assay using the Cobas e411 analyser which employs monoclonal antibodies specifically directed against human TSH. The antibodies labeled with ruthenium complex consist of a chimeric construct from human and mouse-specific components. As a result, interfering effects due to HAMA (human anti-mouse antibodies) are largely eliminated allowing for TSH levels to be determined. The 18-minute assay includes two incubation periods where biotinylated monoclonal TSH specific-antibody, monoclonal TSH-specific antibody and streptavidin coated microparticles are added to the sample and the reagent mixture is aspirated. The microparticles aspirates are coated onto an electrode, and passing a current, results in chemiluminescent emissions from which the final result is determined. TSH level of 0.45–4.2 mU/L (milliunits per litre) is considered normal.

### Qualitative outcomes

Semi-structured interviews will be conducted to collect information regarding the personal experiences of cancer related fatigue, management strategies and patient perceptions of how and why cancer related fatigue affects physical activity levels, including perceived barriers and facilitators. Semi-structured interviewing is the chosen methodology as this form of interviewing utilises open-ended topical questions and allows the participant to expand on the prompts at will thereby allowing various themes and subtopics to emerge naturally [42]. During the interviews, the participant will be able to speak as openly as they wish and provide frank opinions that may address the crux of the issue. Such a format also allows the interviewer the freedom to modify their line of questioning based on the interviewee’s responses with the use of prompts or general encouragement to further elaborate on their experiences/answers [43]. As these interviews will be audio recorded, the tone and inflection of the participant’s voice can indicate their feelings or meanings on the topic discussed which may be extremely useful during the data analysis.

The consolidated criteria for reporting qualitative research (COREQ) 32-item checklist for interviews and focus groups was used to guide the development of the interview and will aid the reporting of the collected results [44]. The interview has been pilot tested on healthy volunteers, to ensure that the questions clearly address the research questions and if all questions can be answered clearly in the time allotted per interview. Each interview will last approximately 45 minutes and allow for some planned and unplanned follow up questions to obtain more detailed answers to questions if needed. To maintain rapport with the patient and minimise time taken on note taking, the interviews will be recorded using two devices and transcribed by P.N. The transcribed data will then be used for the data analysis to present the findings as appropriate. The recording devices will be placed behind or to the side of the patient so that it is not intrusive, allowing the patient and interviewer to focus on the interview. If the interview is conducted virtually due to social distancing restrictions associated with the covid-19 pandemic or situational logistical issues, both audio and video will be recorded using the inbuilt tools of the virtual platform (Microsoft Teams, Zoom or Skype). The interview schedule is shown in S2 File.

### Safety and adverse events

All participants will be evaluated/health screened by the hospital multidisciplinary healthcare team prior to study enrolment. They will undergo health and safety screening to determine their fitness to participate in physical activity testing at all study timepoints. Participants will have their vitals examined, their recent medical history reviewed and undergo a short interview with the lead researcher who will also be conducting the testing. This will ensure that any absolute contraindications to testing will be identified and appropriate next steps are taken i.e., the participant will not be allowed to participate in the testing and will be referred to their primary care physician or consultant for review. If relative contraindications are identified, adequate precautions will be taken but the participant will engage in all quantitative assessments. All testing will be supervised or assisted by the researcher who is also a registered physiotherapist and has the competency to conduct the assessments while minimising the risk of adverse events such as falls etc.

### Analyses

#### Quantitative data

All statistical analyses for the collected quantitative data will be conducted using IBM SPSS Statistics, Version 25.0 (Armonk, NY: IBM Corp.). Statistical assumptions will be checked using standard graphical methods [45]. Sample data will be described using the mean and standard deviation for normally distributed data and median and interquartile range for non-normally distributed data. Changes in outcome measures across time (pre-diagnosis, at the end of treatment, and, 6- and 12-month post-treatment follow-ups) will be analysed using the SPSS MIXED procedure. The best fitting covariance matrix will be identified by that which minimises the Hurvich and Tsai’s criterion. Post-hoc pairwise comparisons with Sidak-adjusted p values will be conducted where an omnibus test is statistically significant. Statistical significance will be accepted as p < 0.05.

#### Qualitative data

The qualitative data will be analysed by following the procedures outlined for Interpretative Phenomenological Analysis (IPA) by Jonathan Smith [46] as the interview aims to understand the stories and lived experiences of cancer related fatigue of the participants. A particular hallmark of IPA is its commitment to the idiographic; the analytic process will begin with a detailed examination of each case followed by a search for the patterning of responses across cases. The concern is with both convergence and divergence in the analysis. Transcripts will be read line-by-line and analysed by searching for points of descriptive and conceptual note throughout. IPA involves maintaining an open mind and an exploratory attitude in order to produce a comprehensive and detailed account of the findings [47].

The transcript notes will then be transformed into emergent experiential themes that aim to capture the key elements of each participants’ experience which will be framed by the interpretations of the researcher in a clear and concise manner. The interview transcripts and audio recordings will be analysed within the week that it collected. Additional methods to establish the trustworthiness and authenticity of the qualitative research process such as reflexivity and member checking will be utilised [48]. The NVivo software (Release 1.0, QSR International, Burlington USA) will be used for aspects of the data synthesis and analyses [49, 50].

### Reporting of results

Quantitative and qualitative results of this study will be reported across separate manuscripts. The reporting of qualitative results will be guided by the consolidated criteria for reporting qualitative research (COREQ) checklist. PPI involvement in the planning and execution of the study will be reported using the GRIPP2-SF.

## Conclusion

This study will improve the understanding of how cancer related fatigue and physical activity levels change over the first year of HaNC. Additionally, the potential impact of changes in body mass, body composition, sleep quality, physical activity levels, haemoglobin concentration, blood counts, thyroid profile, systemic inflammation, cardiorespiratory fitness, agility as well as upper and lower limb strength on fatigue and physical activity levels will be identified and will therefore help inform power calculations for larger studies in the field. Detailed insight on the patient perceptions of cancer related fatigue and physical activity, will provide important context to the problem of cancer related fatigue. This information will help in the design and planning for powered interventional studies which will investigate the most appropriate management of cancer related fatigue in a timely fashion to improve short term and long-term quality of life.

## Supporting information

S1 File (DOCX)

S2 File (DOCX)
