# Supplementary material for: A protocol for the longitudinal investigation of cancer related fatigue in head and neck cancer with an emphasis on the role of physical activity
Source: PLoS One. 2024 Aug 14;19(8):e0308400. doi: 10.1371/journal.pone.0308400 (PMC11324130; doi:10.1371/journal.pone.0308400)
Supplement: S1 File — (DOCX) [file pone.0308400.s001.docx]

# **Supplemental information – S1**

If absolute contraindications are observed, physical activity testing will not take place, but if only relative contraindications are present, then exercise testing can proceed with caution and safety measures in place. All participants will be health screened prior to commencing any tests.

| Clinical Cancer Complications and Acute Conditions | Contraindications to Exercise Testing and Training |
| --- | --- |
| Factors Related to Cancer Treatment | No exercise on days of intravenous chemotherapy  No exercise before blood draw  Severe tissue reaction to radiation therapy |
| Hematologic | Platelet Count < 50,000  Haemoglobin level < 10.0 g/dL  Absolute Neutrophil Count < 0.5 |
| Musculoskeletal | Bone pain  Severe cachexia (loss of >35% premorbid weight)  Karnofsky performance status score <60%;  Extreme fatigue/Muscle weakness |
| Systemic | Acute infections  Febrile illness: fever > 100 F |
| Gastrointestinal | Severe Nausea  Dehydration  Vomiting or Diarrhoea within 24–36 h |
| Cardiovascular | Chest pain  Resting HR > 100 bpm or < 50 bpm  Resting SBP > 145 mmHg and/or DBP > 95 mmHg  Resting SBP < 85 mmHg  Irregular HR  Swelling of ankles |
| Pulmonary | Dyspnoea  Cough, Wheezing  Chest pain increased by deep breath |
| Neurologic | Ataxia/Dizziness/Peripheral Sensory Neuropathy  Significant decline in cognitive performance  Disorientation  Blurred vision |
