# Supplementary material for: A protocol for the longitudinal investigation of cancer related fatigue in head and neck cancer with an emphasis on the role of physical activity
Source: PLoS One. 2024 Aug 14;19(8):e0308400. doi: 10.1371/journal.pone.0308400 (PMC11324130; doi:10.1371/journal.pone.0308400)
Supplement: S2 File — (DOCX) [file pone.0308400.s002.docx]

# **Supplemental information S2**

## **Interview schedule**

The Head and Neck Cancer Patients will be requested to address the following in the order indicated below. Additional follow-up questions or prompts may be posed to the participants to gather additional details. Up to 60 minutes will be allotted for each participant interview at each of the three timepoints. If any additional details are required, the participant will be followed up via telephone or other forms of virtual meetings such as Skype or Microsoft Teams based on the convenience of the participant. Consent for such follow up measures will be obtained at the time of recruitment.

The interviews will be conducted after the participant has completed their primary treatment, i.e., timepoint 2 (T2) and 11-13 months after recruitment (T4).

Stepwise proforma:

- Introduction of researcher/interviewer
- Describe aims of the interview
- Explain nature of questions, recording equipment, data storage
- Review their consent and remind them that they are free to not answer questions they feel may uncomfortable
- Ask and answer any doubts/questions that the participant may have
- Obtain verbal consent to proceed
- Start recording devices and identify participant using bespoke participant number
- Commence with the interview
- After the interview, check in with the participant and debrief
- Set the next appointment

| Q | Question | Prompt | Additional Probe |
| --- | --- | --- | --- |
| 1 | Can you talk through a typical day when you feel fatigued? | - Can you describe your normal routine for the day how its changed? - What about your activity levels or ability to exercise? | How do you notice the change in your energy levels? i.e., do you feel fatigued all of a sudden or is the onset of fatigue symptoms gradual? |
| 2 | How do your current fatigue levels compare to your fatigue levels before your diagnosis? | - Work - Recreation - Social/family - Do you tend to rest or battle through it as much as you can? | Can you describe how the fatigue feels different?  How do these changes make you feel?  Do you feel the need to plan out your day with respect to PA (work and recreation) to minimise fatigue? |
| 3 | How do you cope with/deal with feeling fatigued day to day? | - Rest, medications? - Music, distraction, imagery? - Carer advice? - Does this help you recover completely? | Has someone talked to you about cancer related fatigue from your cancer team? (What it is, causes, other general information etc.)  If yes, who? |
| 4 | Can you tell me about your physical activity levels before your diagnosis | - What factors affected your participation in PA? |  |
| 5 | What about your physical activity levels after your diagnosis/now? | - What has changed? - Which factors have influenced this? - How has the cancer or cancer treatment changed your activity levels? | Has fatigue affected your PA participation?  Has this been your primary consideration? |
| 6 | There is emerging evidence that says regular physical activity is beneficial during cancer and can help with fatigue. How might this apply to you personally and how do you feel about PA? | - What are your beliefs regarding physical activity during cancer? - Do you worry about participating in physical activity because of the cancer? - Are you currently physically active or participating in any PA? - What about any recreational PA? |  |
| 7 | What can we do to help support you to do more physical activity or exercise? | - Do you have any preferences? / Type of activity - Alone v/s with family v/s group - Home v/s gym or community settings - In relation to fatigue? - Other symptoms? - During treatment - Barriers/facilitators | How would you advise another cancer patient to be physically active? |
| 8 | Is there anything else you wish to add about your fatigue or participating in physical activity? |  |  |
